# A general approach for improving deep learning-based medical relation extraction using a pre-trained model and fine-tuning

**DOI:** 10.1093/database/baz116

**Published:** 2019-12-04

**Authors:** Tao Chen, Mingfen Wu, Hexi Li

**Affiliations:** Department of Computer Science and Engineering, Faculty of Intelligent Manufacturing, Wuyi University, No.22, Dongcheng village, Pengjiang district, Jiangmen City, Guangdong Province, 529020, China

## Abstract

The automatic extraction of meaningful relations from biomedical literature or clinical records is crucial in various biomedical applications. Most of the current deep learning approaches for medical relation extraction require large-scale training data to prevent overfitting of the training model. We propose using a pre-trained model and a fine-tuning technique to improve these approaches without additional time-consuming human labeling. Firstly, we show the architecture of Bidirectional Encoder Representations from Transformers (BERT), an approach for pre-training a model on large-scale unstructured text. We then combine BERT with a one-dimensional convolutional neural network (1d-CNN) to fine-tune the pre-trained model for relation extraction. Extensive experiments on three datasets, namely the BioCreative V chemical disease relation corpus, traditional Chinese medicine literature corpus and i2b2 2012 temporal relation challenge corpus, show that the proposed approach achieves state-of-the-art results (giving a relative improvement of 22.2, 7.77, and 38.5% in F1 score, respectively, compared with a traditional 1d-CNN classifier). The source code is available at https://github.com/chentao1999/MedicalRelationExtraction.

## Introduction

Medical relations, such as chemical disease relations (CDRs) and chemical protein relations in modern medicine, herb-syndrome relations and formula-disease relations in traditional medicine, play a key role in a number of biomedical-related applications, e.g. clinical decision-making, drug discovery and drug side-effect detection. Manually extracting these relations is difficult and time-consuming. With recent rapid increases in the scale of biomedical texts and literature, the automatic extraction of meaningful medical relations has received increasing attention over the past decade (1). Relation extraction is usually considered as a classification problem. Three kinds of approaches have been applied to extract medical relations: rule-based approaches (2, 3), shallow machine learning approaches (4, 5) and deep learning approaches (1, 6).

Rule-based approaches require domain experts to define heuristic rules to target a special task (7). Shallow machine learning approaches consider medical relation extraction as a classification problem and generally use supervised learning and feature engineering to obtain high performance. These approaches require manually constructed features or rules. Deep learning approaches use neural networks to automatically capture the syntactic and semantic features of the text without feature engineering. Among current deep learning approaches, convolutional neural networks (CNNs) are one of the key drivers of improvements (8).

However, most deep learning approaches for medical relation extraction are supervised and thus require large-scale training data to prevent overfitting of the training model. Typically, at least 5000 labeled data per category are needed for acceptable performance, and more than 10 million are required to match or exceed human performance (9). Although many medical relation extraction corpora have been created over recent years, most of them are too small to train a deep neural network, especially to train the neural networks which have achieved success in the computer vision or natural language processing (NLP) domains. Munkhdalai *et al.* (10) compared shallow machine learning approaches with deep learning approaches for clinical relation identification, and found that the shallow form remains advantageous over deep learning for clinical relation identification, although deep learning models demonstrate the potential for significant improvement if more training data were available. Huynh *et al.* (11) concluded that more complex CNN variants, such as convolutional recurrent neural networks and CNNs with attention, perform worse than traditional CNNs. We think this is because the corpus they used only contained several thousand labeled training data, and complex models are more likely to cause overfitting with such limited datasets.

Though it is expensive to collect large amounts of training data for the medical domain, a large number of unstructured clinical records or biomedical texts and literature are created every day. Wang and Fan (12) proposed the integration of unlabeled data to help solve the overfitting problems that occur when there are insufficient labeled data. Many methods have been developed to take advantage of unstructured data, such as training domain-specific word embeddings, transfer learning and fine-tuning of pre-trained models. Model pre-training and fine-tuning take a model that has already been trained for a given task and applies it to a second, similar task. This takes advantage of the features extracted on the first task without training from scratch on the second task. Thus, it is a kind of inductive transfer learning (13), which was initially used on a large scale in the field of computer vision (14–16). In text mining or NLP, typical pre-training approaches include Embeddings from Language Models (ELMo) (17), Universal Language Model Fine-tuning (ULMFiT) (13), Bidirectional Encoder Representations from Transformers (BERT) (18), OpenAI Generative Pre-training Transformer (GPT) (19) and GPT-2 (20).

In this paper, we focus on pre-training models from unstructured text and fine-tuning the pre-trained models to improve the performance of existing deep learning-based medical relation extraction approaches with limited training data. First, we show the architecture of BERT, which is a novel and effective approach for pre-training models on large-scale unstructured text. Then, we use a one-dimensional convolutional neural network (1d-CNN) to fine-tune the pre-trained BERT model for medical relation extraction. To evaluate our approach, extensive experiments are conducted on three kinds of real-world medical relation extraction datasets in different languages: the BioCreative V CDR corpus (21–24), traditional Chinese medicine (TCM) literature corpus (25), and i2b2 2012 temporal relations challenge corpus (26, 27). The proposed approach achieves state-of-the-art results on all three datasets (giving a relative improvement of 22.2, 7.77 and 38.5% in F1 score, respectively, compared with a traditional 1d-CNN classifier). To the best of our knowledge, this is the first general-purpose approach to achieve state-of-the-art performance in all three medical relation extraction tasks. The results can be used in applications including chemical-disease interactions research, poly-pharmacology research and adjuvant clinical treatment. The source code is available at https://github.com/chentao1999/MedicalRelationExtraction. We have made a web service of our system available at http://120.78.238.14:8080/cdr.

## Materials and methods

### Data sources

**Figure 1:**
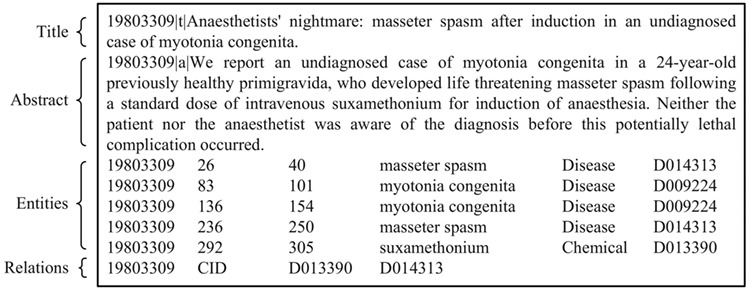
An example of the BC5CDR corpus (PubTator format, PMID:19803309).

**Figure 2:**
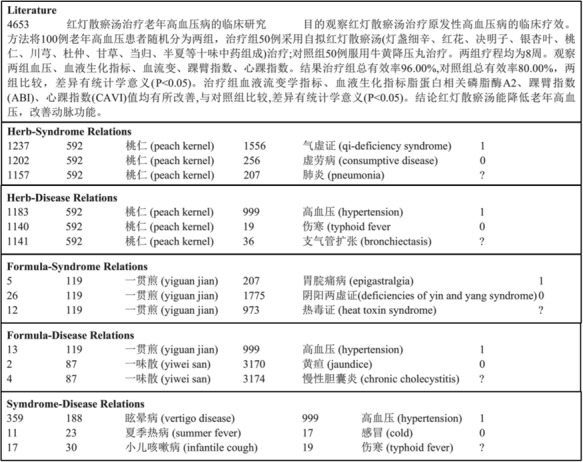
An example of the TCM corpus.

**Figure 3:**
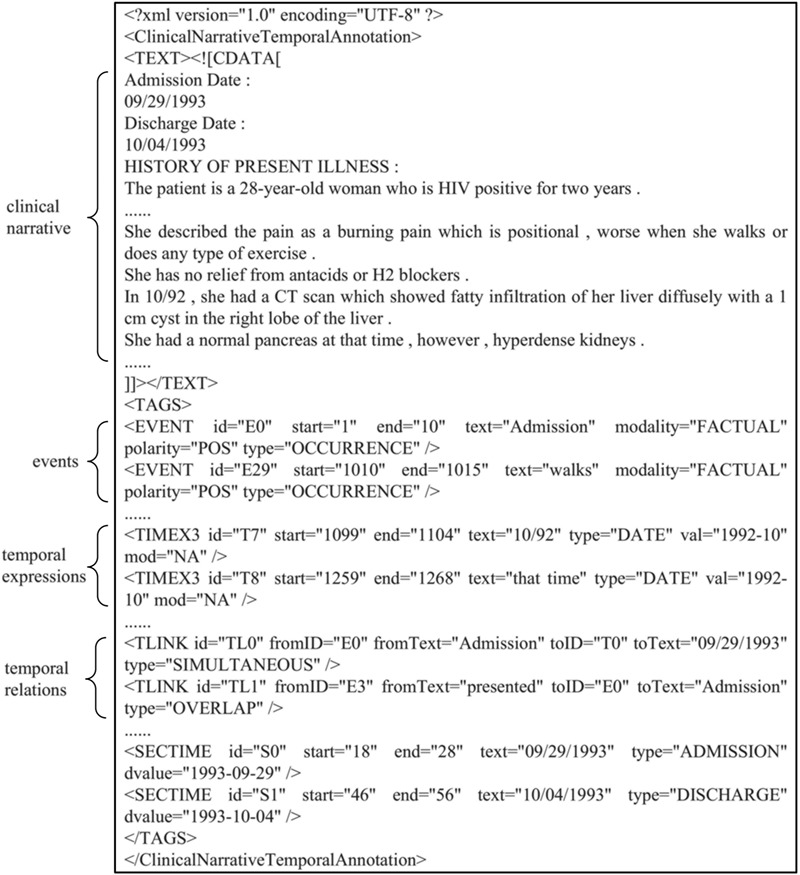
Sample text excerpt of the i2b2 temporal corpus.

The proposed method is a general-purpose approach. In this work, we utilize three different kinds of medical relation extraction corpora:

1. BioCreative V CDR task corpus (in short, BC5CDR corpus) (21–24): this consists of 1500 PubMed articles with 4409 annotated chemicals, 5818 diseases and 3116 chemical-disease interactions. Figure 1 shows a PubTator format (tab-delimited format) file for the article (PMID: 19803309) in the training set of the corpus. A summary of this corpus is presented in Table 1. The relation task data are publicly available through BioCreative V at https://biocreative.bioinformatics.udel.edu/resources/corpora/biocreative-v-cdr-corpus/.
2. TCM literature corpus (in short, TCM corpus) (25): the abstracts of all 106,150 papers published in the 114 most popular Chinese TCM journals from 2011 to 2016 are collected, including details of 3024 herbs, 4957 formulae, 1126 syndromes and 1650 diseases. Five types of relations are annotated. Figure 2 gives an example of TCM literature and relations in the corpus. The statistics of the corpus are summarized in Table 2. The negative relations are for co-occurring entities that do not have an explicit relation. The unlabeled relations are for co-occurring entities that their relations are not annotated by TCM experts. Only 10% of the candidate relations are annotated. The entire dataset is available online at http://arnetminer.org/TCMRelExtr.
3. The 2012 informatics for integrating biology and the bedside (i2b2) project temporal relations challenge corpus (in short, i2b2 temporal corpus) (26, 27): This contains 310 de-identified discharge summaries of over 178,000 tokens, with annotations of clinically significant events, temporal expressions and temporal relations in clinical narratives. On average, each discharge summary in the corpus contains 86.6 events, 12.4 temporal expressions and 176 raw temporal relations. In this corpus, eight kinds of temporal relations between events and temporal expressions are defined: BEFORE, AFTER, SIMULTANEOUS, OVERLAP, BEGUN_BY, ENDED_BY, DURING and BEFORE_OVERLAP. Figure 3 shows an excerpt of a patient report and its annotation of events, temporal expressions and temporal relations in the training set of the corpus. We present the details of this corpus in Table 3. The annotations are available at http://i2b2.org/NLP/DataSets.

**Table 1:** Summary of the BioCreative V CDR corpus

Dataset	Articles	Chemical mention (ID)	Disease mention (ID)	CID relation
Training	500	5203 (1467)	4182 (1965)	1038
Dev	500	5347 (1507)	4244 (1865)	1012
Test	500	5385 (1435)	4424 (1988)	1066

From the above tables, it is clear that BC5CDR and the TCM corpus are annotated with two categories, and there are hundreds of labeled data per category. The i2b2 temporal corpus is much larger. There are 33,635 annotated samples across eight kinds of temporal relations, an average of 4204.4 labeled data per category. None of these corpora is large enough (<5000 labeled data per category) to train a deep neural network for acceptable performance.

### Overview of our approach

**Figure 4:**
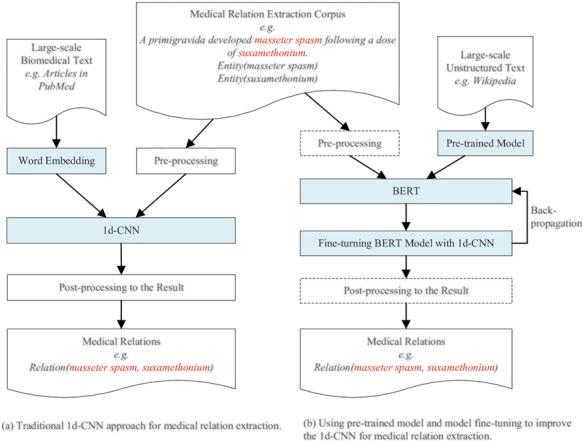
The architecture of the traditional approach and our proposed approach.

An overview of our approach for improving deep learning-based medical relation extraction using a pre-trained model and model fine-tuning is shown in Figure 4. The left sub-figure (Figure 4a) shows the architecture of the traditional approach using a 1d-CNN model to classify medical relations. The word embeddings and pre-processed medical relation extraction corpus are the input of the 1d-CNN model. The word embeddings are real number vector representations of words or phrases from the vocabulary. It is usually trained from large-scale unstructured biomedical text, literature or clinical records for medical relation extraction.

The right sub-figure (Figure 4b) is the architecture of our proposed approach. ‘Pre-trained Model’ refers to binary checkpoint files in which the neural network architecture, weights and variables are stored. ‘BERT’ refers to a kind of pre-training approach. Before fine-turning begins, ‘BERT’ restores its variables and neural network parameters by loading the ‘Pre-trained Model’ files. After BERT loads the pre-trained model, fine-tuning begins. The training error back-propagates to BERT to fine-tune its parameters, which are firstly restored from the pre-trained model. Our approach also uses a 1d-CNN model to classify medical relations. The main differences between our proposed approach and the traditional approach are:

1. We use a pre-trained model instead of word embeddings as the input of the 1d-CNN model.
2. We combine the 1d-CNN with BERT and use the 1d-CNN to fine-tune the parameters of the BERT model. The operations in the dashed rectangle, such as pre-processing and post-processing, are optional in our experiments. Our proposed approach is an end-to-end general-purpose. We didn’t perform pre-processing and post-processing in all three experiments. Experimental results show that our approach achieves state-of-the-art performance even without pre-processing the corpus and post-processing the results. Other researchers who use our method can add pre-processing and post-processing for a special corpus to further improve performance.
3. The model fine-tuning process is realized by back-propagating the training error of the 1d-CNN to the BERT model. This is a dynamic model training process. Traditional approaches that concatenate embeddings with the input at different layers still train the main task model from scratch and treat pre-trained embeddings as fixed parameters, limiting their usefulness (13).

The following sections describe the pre-trained model and our approach in detail and explain how to use the 1d-CNN to fine-tune the pre-trained BERT model for medical relation extraction.

**Table 2:** Summary of the TCM corpus

Relation type	Labeled relations		Unlabeled relations
	Positive	Negative	
Herb-syndrome	538	582	10 077
Herb-disease	534	642	10 579
Formula-syndrome	392	574	8693
Formula-disease	377	411	7094
Syndrome-disease	431	532	8681

### Pre-trained model

Most supervised learning approaches on task-specific datasets are brittle and sensitive to slight changes in the data distribution (28) and task specification (29). Pre-trained models are usually trained by a pre-training approach in an unsupervised way on large-scale unstructured general-domain text (like Wikipedia) on GPU cluster or cloud TPUs for several days. They are general systems that can be used as components in many downstream tasks.

**Table 3:** Summary of the i2b2 temporal relation corpus

	Training set	Test set
Discharge summaries	190	120
Events	16,468	13,594
Temporal expressions	2,366	1,820
Temporal relations	33,635	27,736
BEFORE	13,467	10,789
AFTER	2,211	1,941
SIMULTANEOUS	4,725	4,142
OVERLAP	7,061	4,877
BEGUN_BY	996	788
ENDED_BY	797	688
DURING	1,037	875
BEFORE_OVERLAP	3,249	3,636
Unlabeled	92	0

As mentioned above, typical pre-training approaches include ELMo, ULMFiT, GPT, GPT-2 and BERT. ELMo uses a bidirectional long short-term memory (LSTM) to pre-train a bidirectional language model (biLM) on a large text corpus. Once pre-trained, the biLM can compute representations for downstream tasks (17). ULMFiT uses a three-layer LSTM architecture to pre-train an ImageNet-like language model and uses a discriminative fine-tuning technique to allow different layers to capture different types of information (13). GPT (19) uses a left-to-right architecture, where every token can only attend to previous tokens in the self-attention layers of the transformer (30). GPT-2 largely follows the details of the GPT model, but has over an order of magnitude more parameters than the original GPT (20). BERT uses a deep bidirectional transformer that is jointly conditioned on both the left and right contexts in all layers to pre-train masked language models (18).

In this work, we use BERT as our pre-training approach because (i) it achieves better performance than most other pre-training approaches (ELMo, OpenAI GPT, etc.) in many NLP tasks (18) and (ii) it is open source. Pre-training is fairly expensive. Several pre-trained BERT models are available at https://github.com/google-research/bert. We will never need to pre-train our own model from scratch.

An illustration of the architecture of BERT is shown in Figure 6. BERT uses WordPieces (31) as tokens rather than words. Consider the following example sentence pair:

**Example 1:** suxamethonium_[D013390]_ masseter spasm_[D014313]_ a dose-response study*.*

**Figure 5:**
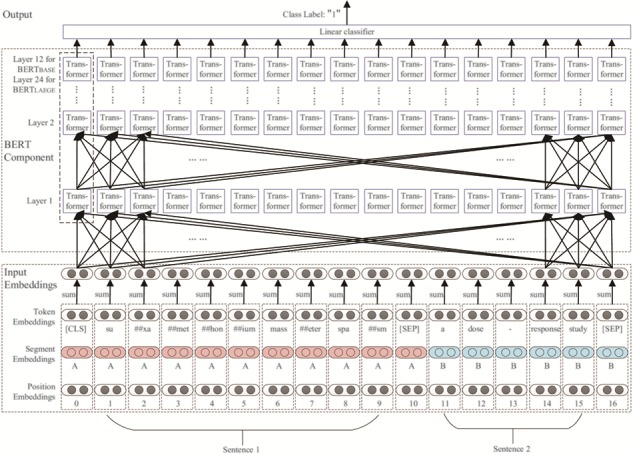
An illustration of the architecture of BERT (18).

This is broken down into smaller chunks, as shown in the Token Embeddings line in Figure 5. ‘##’ refers to the split word pieces. For example, suxamethonium is split into five tokens: su, ##xa, ##met, ##hon and ##ium. In the Token Embeddings, [CLS] refers to a special classification embedding. [SEP] refers to the end of a sentence. In the Segment Embeddings, A (in red) refers to the first sentence of the sentence pair and B (in blue) refers to the second sentence of the sentence pair. The Position Embeddings refers to the serial number of tokens in the sentence pair sequence. The input embeddings are constructed by summing the values of the corresponding token, segment and position embeddings (18). The i-th value of the input embedding is computed as follows:

(1) \begin{equation*} {v}_{input}^{(i)}={v}_{token}^{(i)}+{v}_{segment}^{(i)}+{v}_{position}^{(i)} \end{equation*}

where *v*^(*i*)^ refers to the *i*-th value of an embedding; *v_input_*, *v_token_* and *v_segment_* refer to the input embeddings, token embeddings, segment embeddings and position embeddings, respectively.

The architecture of BERT is basically a multi-layer transformer encoder stack. In each layer, the number of transformer encoder nodes is equal to the length of the input embedding. Each node fully connects with every transformer encoder node in the upper layer. The basic BERT model has 12 layers, whereas the large BERT model has 24 layers. Each transformer encoder node has two sub-layers: (i) a self-attention layer, which helps the encoder look at other words in the input sentence as it encodes a specific word and (ii) a position-wise fully connected feed-forward network, which receives the output of the self-attention layer (19).

To pre-train a language model, BERT uses two novel unsupervised prediction tasks: (i) masked language model, in which several tokens in each sequence are randomly erased and replaced with a special token (‘masked’). A model is trained by using the unmasked words to predict the masked word. (ii) Next sentence prediction, where 50% of sentence pairs are labeled with ‘IsNext’, and the other 50% sentence pairs have the second sentence randomly replaced and the whole sentence pair labeled with ‘NotNext’. A model is trained with both ‘IsNext’ and ‘NotNext’ sentence pairs.

After random dropout regularization, the output of the transformer encoder stack is connected with a linear classifier for sequence classification training. The training error is back-propagated to the first layer of the pre-trained BERT model to realize the model fine-tuning process.

### A general approach for medical relation extraction

We formulated the medical relation extraction task as a classification problem that judges whether a given pair of medical entities (e.g. chemical and disease) was asserted with an induction relation in the article. Our approach is a general-purpose approach. The ‘relation’ here depends on the relation defined in the specific corpus. It can refer to the existence of a relation (in BC5CDR corpus and TCM corpus) or a specific type of relation (in i2b2 temporal corpus). In BC5CDR and TCM corpus, the relation is undirected. In i2b2 temporal corpus, it is directed, because time has a direction.

As a classification problem, the specific input of our model is the two entities in a relation (see sentence 1 in Figure 5) and the context entities co-occurred with (see sentence 2 in Figure 5). The output is the existence of a relation (for BC5CDR corpus and TCM corpus) or the label of a specific type of relation (for i2b2 temporal corpus). Take the following data from the BC5CDR corpus as an example:

**Example 2:** 1601297|a|The electrocardiograms (ECG) of 99 cocaine-abusing patients were compared with the ECGs of 50 schizophrenic controls. Eleven of the cocaine abusers and none of the controls had ECG evidence of significant myocardial injury defined as myocardial infarction, ischemia and bundle branch block.

**Table:** 

1601297	33 50	Myocardial injury		Disease	D009202
1601297	83	90	Cocaine	Chemical	D003042
1601297	135	142	Cocaine	Chemical	D003042
1601297	194 207	Schizophrenic		Disease	D012559
1601297	232	239	Cocaine	Chemical	D003042
1601297	305 322	Myocardial injury		Disease	D009202
1601297	334 355	Myocardial infarction		Disease	D009203
1601297	357	365	Ischemia	Disease	D007511
1601297	371 390	Bundle branch block		Disease	D002037
1601297	CID	D003042	D009203		
1601297	CID	D003042	D002037		

This is a PubTator format (tab-delimited format) file for the article (PMID: 1601297) in the training set of the BC5CDR corpus. The first two lines are the title and the abstract of the annotated article. There are one chemical (MeSH ID: D003042), five disease (MeSH ID: D009202, D012559, D009203, D007511, and D002037) and two CID relations (<D003042, D009203> and < D003042, D002037>). For CID relation <D003042, D009203>, we concatenate the chemical ‘cocaine’ and disease ‘myocardial infarction’ as the first sentence and concatenate the title and abstract as the second sentence. The first and second sentences form a sentence pair sequence, which is input into the BERT model.

Using a sentence pair as the input of a neural network is an effective mechanism to model the relationship between two sentences. It is commonly used in many NLP tasks (e.g. language model (32), machine translation (33) and natural language inference (34)). Just like the shallow machine learning methods, different features are concatenated into a long vector as the input of the classifier (e.g. SVM, CRF and Naive Bayes). For an end-to-end neural network system, it is easy and intuitive to concatenate different kinds of information provided by the training data into a sequence as the input of the system.

In our approach, we concatenate the entities as the first sentence input and concatenate the title and abstract as the second sentence input, just because BERT needs a sentence pair as input, and this is an effective mechanism to model the relationship between entities and their context. A ‘sentence’ here can be an arbitrary span of contiguous text, rather than an actual linguistic sentence.

The relations in the BC5CDR dataset are annotated at the abstract level, and only entity pairs which have CID relation are annotated. Following the participating systems of the BioCreative V Chemical Disease Relation (CDR) Task (8, 21), we label these annotated entity pairs with ‘1’ to generate positive training samples. Then, we randomly select a ‘chemical’ entity and a ‘disease’ entity in a sample document to make an entity pair. If the entity pair is not annotated, we think the two entities in this entity pair have no CID relation. We label this entity pair with ‘0’ to generate a negative sample. Positive and negative samples are generated according to the ratio of 1:1 in order to train a balanced model to predict new relations.

For entity pair <D003042, D009203>, a positive sample is generated as follows:

‘Id_1 cocaine myocardial infarction\tElectrocardiographic evidence of myocardial injury in psychiatrically hospitalized cocaine abusers. The electrocardiograms (ECGs) of 99 cocaine-abusing patients were compared with the ECGs of 50 schizophrenic controls. Eleven of the cocaine abusers and none of the controls had ECG evidence of significant myocardial injury defined as myocardial infarction, ischemia and bundle branch block.’

For entity pair <D003042, D002037>, another positive sample is generated as follows:

‘Id_2 cocaine bundle branch block\tElectrocardiographic evidence of myocardial injury in psychiatrically hospitalized cocaine abusers. The electrocardiograms (ECGs) of 99 cocaine-abusing patients were compared with the ECGs of 50 schizophrenic controls. Eleven of the cocaine abusers and none of the controls had ECG evidence of significant myocardial injury defined as myocardial infarction, ischemia and bundle branch block.’

We randomly select two diseases in (D009202, D012559 and D007511) to generate two entity pairs that have no CID relation and use them to generate two negative samples. They may be like this:

‘Id_3 cocaine myocardial injury block\tElectrocardiographic evidence of myocardial injury in psychiatrically hospitalized cocaine abusers. The electrocardiograms (ECGs) of 99 cocaine-abusing patients were compared with the ECGs of 50 schizophrenic controls. Eleven of the cocaine abusers and none of the controls had ECG evidence of significant myocardial injury defined as myocardial infarction, ischemia and bundle branch block.

Id_4 cocaine schizophrenic\tElectrocardiographic evidence of myocardial injury in psychiatrically hospitalized cocaine abusers. The electrocardiograms (ECG) of 99 cocaine-abusing patients were compared with the ECGs of 50 schizophrenic controls. Eleven of the cocaine abusers and none of the controls had ECG evidence of significant myocardial injury defined as myocardial infarction, ischemia and bundle branch block.’

In the TCM corpus, both positive and negative samples are annotated. We use the original data to train a model.

If there are multiple relation categories, as it is in the i2b2 temporal corpus, each pair of entities (including an event entity and a temporal expression entity) is labeled with one of the eight kinds of temporal relations (BEFORE, AFTER, SIMULTANEOUS, OVERLAP, BEGUN_BY, ENDED_BY, DURING and BEFORE_OVERLAP). We also use the original samples directly to train a multi-class classifier for prediction.

### Fine-tuning the BERT model with 1d-CNN

In the original BERT model, a linear classifier is used to fine-tune the pre-trained model for sequence classification. In this work, to improve the performance of the 1d-CNN in medical relation extraction tasks, we use the multi-filter 1d-CNN classifier (in short, 1d-CNN) proposed by Kim (35) to fine-tune the pre-trained BERT model.

The training object of the 1d-CNN is to minimize the ranking loss below:

(2) \begin{equation*} \sum_{d\in T}\max \left\{0,1-g(d)+g\left({d}^{\prime}\right)\right\} \end{equation*}

where *d* is a medical relation document in training set *T* with a positive label; ${d}^{\prime }$ is another document in *T* with a negative label; $g(\cdotp )$ is the scoring function that represents the 1d-CNN architecture and $g(d)$ and $g({d}^{\prime})$ are the scores of positive and negative documents, respectively. The training procession of the 1d-CNN classifier is to make $g(d)$ approximately 1 and $g({d}^{\prime})$ approximately 0.

Positive label and negative label documents are the positive and negative samples in a corpus that is used to train a binary classifier. When there are multiple chemicals, diseases and relations in a document, multiple entity pairs are generated, and each pair of entities share the same context document. One entity pair and its context form a positive sample. For the BC5CDR corpus, we randomly select two entities that have no medical relation to generate negative samples. For the TCM and i2b2 temporal corpus, we use the original annotated data to train a model.

The main architecture of the 1d-CNN consists of (i) an input layer, which converts variable-length medical relation documents into fixed-length vectors; (ii) a convolution layer, in which multiple filters move across the input vectors to extract semantic features through one-dimensional convolution; (iii) a pooling layer, in which the most useful semantic features are selected by a max-overtime pooling operation (36) and (iv) an output layer, in which multiple features are concatenated and classified by a fully connected SoftMax classifier. In the training process, the training error is back-propagated to fine-tune the parameters of the BERT, which are firstly restored from the pre-trained model.

Pre-trained BERT models can also be used in other deep learning methods, such as RNN and LSTM. However, they cannot be used as features in these methods because the pre-trained BERT models are neural networks with pre-trained parameters, not a vector with numerical values. They are usually used as components of other neural networks.

## Experiments and results

### Measures

For all three corpora, we use the precision, recall and F1 measure as evaluation metrics for the medical relation extraction performance. The precision, recall, and F1 measure are computed as follows:

(3) \begin{equation*} \mathrm{Precision}\left(\mathrm{P}\right)=\frac{TP}{TP+ FP} \end{equation*}

(4) \begin{equation*} \mathrm{Recall}\left(\mathrm{R}\right)=\frac{TP}{TP+ FN} \end{equation*}

(5) \begin{equation*} \mathrm{F}1=\frac{2\times P\times R}{P+R} \end{equation*}

where *TP* denotes true positive, *FP* denotes false positive and *FN* denotes false negative in the confusion matrix.

Medical relation extraction in the i2b2 2012 temporal relations challenge is a multi-class classification task. In this work, we use the evaluation scripts (Available at http://i2b2.org/NLP/DataSets) provided by the challenge organizer to evaluate the performance of our approach on the i2b2 temporal corpus.

### Experimental settings

For all three corpora, we use a PyTorch implementation of Kim’s 1d-CNN (Software available at https://github.com/wabyking/TextClassificationBenchmark). For the pre-trained word embeddings used in 1d-CNN, we use two English embeddings (Glove embeddings (37) and PubMed embeddings (Available at http://bio.nlplab.org/)) and two Chinese embeddings (general embeddings (38) and TCM literature embeddings trained by ourselves) for the English and Chinese corpora, respectively.

For the Glove embeddings (Available at https://nlp.stanford.edu/projects/glove/), we use 300-dimensional vectors trained on 6B tokens from Wikipedia 2014 and Gigaword 5. For general Chinese embeddings (Available at https://github.com/Embedding/Chinese-Word-Vectors), we use the 300-dimensional vectors trained on a mixed corpus including Baidu Encyclopedia, Chinese Wikipedia, People’s Daily News and similar. For TCM literature embeddings, we use the traditional Chinese medicine literature in the TCM corpus. This contains 15M words, and the vocabulary size is 57K. We use word2vec tools (Available at https://code.google.com/archive/p/word2vec/)to train the vectors. The embeddings have 300 dimensions.

To train 1d-CNN, we concatenate the two entities in one relation and the text they co-occurred in as a long document and use this document as the input of 1d-CNN. We use rectified linear units for the activation function, filter windows of lengths 3, 4 and 5 with 100 feature maps each, an AdaDelta decay parameter of 0.95 and a dropout rate of 0.5 on all three corpora. The maximum sequence length is set to 400 for the BC5CDR corpus, 300 for the TCM corpus and 1000 for the i2b2 temporal corpus.

To fine-tune 1d-CNN using the pre-trained BERT model, we concatenate the two entities in one relation as one input sentence and the text in which the two entities co-occurred as the other input sentence. We use the ‘uncased_L-12_H-768_A-12’ model for the English corpus and the ‘chinese_L-12_H-768_A-12’ model for the Chinese corpus. Both models are pre-trained on the BERT-Base network, which has 12 layers, 768 hidden nodes, 12 heads and 110M parameters. As reported by Devlin *et al.* (2), the ‘uncased_L-12_H-768_A-12’ model is trained on BooksCorpus (800M words) (39) and English Wikipedia (2500M words). We use a learning rate of 5 × 10^−5^.

We trained our models using a single NVIDIA GeForce GTX 1080Ti GPU with 12 GB of RAM. Fine-tuning the pre-trained BERT model on GPUs with 12–16 GB of RAM may cause out-of-memory issues (https://github.com/google-research/bert#out-of-memory-issues). The factors that affect memory usage are the maximum sequence length and the batch size for training. We used maximum sequence lengths of 200, 110 and 170 and training batch sizes of 20, 32 and 8 for the BC5CDR corpus, TCM corpus and i2b2 temporal corpus, respectively. For the other parameters, we use the default settings for 1d-CNN and BERT.

### BioCreative V CDR task

Table 4 presents the system results achieved on the BC5CDR corpus. The best results are highlighted in boldface. The BioCreative V CDR task has two subtasks: disease named entity recognition (DNER) and chemical-induced disease (CID) relation extraction. In Table 4, the ‘best system of BioCreative V CDR extraction challenge’ uses the chemical and disease entities automatically recognized in the DNER subtask, whereas the other four approaches use the gold annotated entities as the input for the CID relation extraction task.

**Table 4:** System results on the BC5CDR corpus

Approach	Precision	Recall	F1
Best system of BioCreative V CDR extraction challenge (34)	0.5567	0.5844	0.5703
1d-CNN with gold entity annotation (Glove Embeddings)	0.6085	0.5642	0.5855
1d-CNN with gold entity annotation (PubMed embeddings)	0.7439	0.5699	0.6454
Pons *et al.* (37)	0.731	0.676	0.7020
BERT with pre-trained model	0.7493	0.6673	0.7059
1d-CNN fine-tuning the pre-trained BERT model	**0.7505**	**0.6838**	**0.7156**

The best results for each metric are highlighted in boldface.

The 1d-CNN approach with general embeddings (Glove embeddings) achieves an F1 score of 0.5855. Using domain-specific embeddings (PubMed embeddings) improves the 1d-CNN F1 score to 0.6454, a relative improvement of 10.2%. Pons *et al.* (40) refers to the SVM approach with rich human-engineered features proposed by Pons *et al.* Using the gold annotated entities and post-challenge features, this approach achieves an F1 score of 0.7020. ‘BERT with pre-trained model’ refers to the original BERT model in which a linear classifier is used to fine-tune the pre-trained model. The ‘1d-CNN fine-tuning BERT with pre-trained model’ refers to our proposed approach. It achieves state-of-the-art performance without any human-engineered features or pre/post-processing operations, giving a relative improvement of 1.9% compared to Pons *et al.* and 10.8% compared to 1d-CNN with PubMed embeddings. These results validate the influences of the pre-trained model and the fine-tuning technique in terms of medical relation extraction.

There are 14,901 words found in the vocabulary of the BC5CDR corpus. Among these, 11,652 words were found in the Glove embeddings and 13,928 words were found in the PubMed embeddings. BERT split these words into 9175 sub-word units (tokens), with all word pieces included in the vocabulary of the pre-trained BERT model. The experimental results show that higher word coverage can improve the recall of the approach.

### Relation extraction task using TCM literature

Table 5 presents the system results achieved on the TCM literature corpus. HS, HD, FS, FD and SD refer to the herb-syndrome, herb-disease, formula-syndrome, formula-disease and syndrome-disease relations in TCM, respectively. The top three results of the compared systems, i.e. Basic SVM, Iterative SVM and HFGM, were reported by Wan *et al.* (25). HFGM is the abbreviation of the heterogeneous factor graph model, which is a unified graphical model proposed by Wan *et al.* It is used to simultaneously infer the labels of all the candidate relations by employing the concept of collective inference (25). ‘1d-CNN general embeddings’ refers to the 1d-CNN approach using general Chinese embeddings as input, ‘1d-CNN TCM embeddings’ refers to the 1d-CNN approach using TCM literature embeddings as input, ‘BERT with pre-trained model’ refers to the original BERT model in which a linear classifier is used to fine-tune the pre-trained model and ‘1d-CNN fine-tuning’ refers to the 1d-CNN approach using the basic BERT network as its component and fine-tuning the pre-trained BERT model with 1d-CNN. Following the work of Wan *et al.* (25), we perform a five-fold cross-validation to evaluate the performance of our model. The best results for each relation type and metric are highlighted in boldface.

**Table 5:** System results on the TCM corpus

Approach	Metric	HS	HD	FS	FD	SD	Average
Basic SVM	Precision	0.7889	0.7913	0.8012	0.8104	0.7772	0.7930
	Recall	0.7234	0.7459	0.7232	0.7308	0.7322	0.7315
	F1	0.7547	0.7679	0.7602	0.7685	0.7540	0.7609
Iterative SVM	Precision	0.8335	0.8310	0.8433	0.8555	0.8188	0.8354
	Recall	0.7766	0.7951	0.7775	0.7841	0.7834	0.7836
	F1	0.8040	0.8127	0.8091	0.8182	0.8007	0.8087
HFGM	Precision	0.9094	0.8948	0.9081	0.9107	0.8987	0.9039
	Recall	**0.8693**	0.8734	**0.8569**	0.8825	0.8687	0.8698
	F1	0.8889	0.8840	**0.8818**	0.8964	0.8786	0.8856
1d-CNN general embeddings	Precision	**0.9670**	0.9264	**0.9572**	0.9041	**0.9982**	0.9505
	Recall	0.7460	0.9070	0.6937	0.7950	0.8150	0.7913
	F1	0.8422	0.9166	0.8045	0.8461	0.8973	0.8613
1d-CNN TCM embeddings	Precision	0.9382	0.9365	0.8984	0.9150	0.9784	0.9333
	Recall	0.7451	0.9044	0.7059	0.8249	0.8463	0.8053
	F1	0.8306	0.9201	0.7906	0.8677	0.9076	0.8633
BERT with pre-trained model	Precision	0.9263	0.9675	0.8885	0.9570	0.9589	0.9319
	Recall	0.8457	0.9440	0.8334	0.9281	0.9237	0.8901
	F1	0.8842	0.9556	0.8601	0.9423	0.9410	0.9105
1d-CNN fine-tuning	Precision	0.9365	**0.9745**	0.8974	**0.9702**	0.9806	**0.9518**
	Recall	0.8629	**0.9553**	0.8350	**0.9352**	**0.9420**	**0.9061**
	F1	**0.8982**	**0.9648**	0.8651	**0.9522**	**0.9609**	**0.9282**

The best results for each metric are highlighted in boldface.

As we can see, almost all the algorithms have very high performance on this corpus. This task is easier than the other two tasks because it is a binary classification problem, and the relations in this corpus are annotated at the instance level.

The ‘1d-CNN fine-tuning’ approach achieves the best performance on four of the five relation types, outperforming the others by a good margin on the herb-disease, formula-disease and syndrome-disease relations. Compared with *HFGM*, which is a probability graph model specially designed for TCM relation extraction problems, ‘1d-CNN fine-tuning’ improves the F1 score by 1.04, 9.14, 6.22 and 9.37% on the herb-syndrome, herb-disease, formula-disease and syndrome-disease relations, respectively. This indicates that our proposed approach is effective for TCM relation extraction.

In formula-syndrome relations, the ‘1d-CNN fine-tuning’ approach achieves an F1 score that is 1.67% lower than that of HFGM. This may be because we only find 197 out of the 966 annotated formula-syndrome relations that co-occurred in the literature of the TCM corpus, whereas HFGM uses all 966 annotated formula-syndrome relations (see Table 2). In TCM corpus, the format of annotated relation documents is: ‘ID\tEntityID1\tEntityName1 \tEntityID2\tEntityName2\tLabel’, and the TCM literature which is used to annotate TCM relations is in a separate document. We need to search for the context of the relations in the literature document. As shown in Table 2, there are 966 annotated formula-syndrome relations, including 392 positive relations and 574 negative relations. When we searched for the context that the entities of a formula-syndrome relation co-occurred in, we only found 197 relations their entities co-occurred in the literature of the TCM corpus.

For the other four kinds of relations, the numbers are 256/1120 (herb-syndrome relations), 1176/1176 (herb-disease relations), 788/788 (formula disease relations) and 268/964 (syndrome-disease relations). Therefore, only 197 formula-syndrome relations are used in our experiment. Our method achieves comparable or better performance with less training data than the comparative methods. It shows the effectiveness of our method.

The ‘1d-CNN with general embedding’ approach achieves the best precision for the herb-syndrome, formula-syndrome and syndrome-disease relations, but has low recall on most relations. The ‘1d-CNN with TCM embedding’ approach has a larger vocabulary coverage than ‘1d-CNN with general embedding’ and achieves slightly better performance. By fine-tuning the pre-trained BERT model, our proposed approach achieves F1 scores of 0.8982, 0.9648, 0.8651, 0.9522 and 0.9609 on the five relations. This corresponds to relative improvements of 6.65, 5.26, 7.53, 12.5 and 7.09% compared with the ‘1d-CNN with general embeddings’ approach, respectively. These results indicate that using the pre-trained model and fine-tuning technique can improve the performance of the 1d-CNN classifier for TCM relation extraction.

### I2b2 2012 challenge clinical temporal relation extraction task

Table 6 presents the system results achieved on the i2b2 temporal relation corpus. ‘1d-CNN with gold entity annotation’ refers to the 1d-CNN approach using annotated gold event and time expressions as entities. ‘Glove embeddings and PubMed embeddings’ refer to the 1d-CNN approach using Glove embeddings and PubMed embeddings as initial input, respectively. ‘The best system of i2b2 challenge’ refers to the system that obtained the best performance in the TLINK subtask of the i2b2 2012 temporal relations challenge. This is an SVM classifier-based approach that also uses annotated gold events and time expressions. ‘BERT with pre-trained model’ refers to the original BERT model in which a linear classifier is used to fine-tune the pre-trained model. ‘1d-CNN fine-tuning BERT with pre-trained model’ is our proposed approach.

**Table 6:** System results on the i2b2 temporal corpus

Approach	Precision	Recall	F1
1d-CNN with gold entity annotation (Glove embeddings)	0.4549	0.5846	0.5117
1d-CNN with gold entity annotation (PubMed embeddings)	0.5787	0.5647	0.5716
The best system of i2b2 challenge (27)	**0.71**	0.67	0.6932
BERT with pre-trained model	0.6684	0.7173	0.6920
1d-CNN fine-tuning BERT with pre-trained model	0.6722	**0.7489**	**0.7085**

The best results for each metric are highlighted in boldface.

Note that our proposed approach achieves the best performance, with an F1 score of 0.7085. This represents a relative improvement of 2.21% compared with the best system from the i2b2 challenge. By using a pre-trained model on large-scale unstructured text, our approach achieves a recall of 0.7489, a relative improvement of 11.8% compared with the best system of the i2b2 challenge. Note that our method is a general-purpose end-to-end approach without feature engineering for special tasks. We did not even pre-process the corpus or post-process the classification result in this experiment.

The 1d-CNN approach with general embeddings (Glove embeddings in the table) achieves an F1 score of 0.5117. Using domain-specific embeddings (PubMed embeddings in the table) improves the F1 score of 1d-CNN to 0.5716. This is a relative improvement of 11.7% compared with the 1d-CNN approach with general embeddings. By fine-tuning the pre-trained BERT model, the F1 score of 1d-CNN improves to 0.7085, a relative improvement of 38.4% compared with the 1d-CNN approach with general embeddings and 23.9% compared with the 1d-CNN approach with domain-specific embeddings. These results indicate that our proposed approach can improve the performance of the 1d-CNN for temporal relation extraction tasks.

## Discussion

In this work, we have presented an approach for chemical disease relation extraction, traditional Chinese medicine literature relation extraction and clinical temporal relation extraction. To the best of our knowledge, this is the first general-purpose approach to achieve state-of-the-art performance in all three medical relation extraction tasks. After performing an in-depth analysis of some specific instances, we found that our proposed approach improves the performance of relation extraction for the following reasons: (i) the pre-trained model is more effective than word embeddings for acquiring useful linguistic knowledge for downstream tasks; (ii) compared with the ‘1d-CNN general embeddings’ approach, our method achieves higher recall on all three corpora. This indicates that fine-tuning the pre-trained model and back-propagating the training error makes better use of the linguistic knowledge acquired from the unstructured text than using pre-trained embeddings as input and training the main task model from scratch. Treating pre-trained embeddings as fixed parameters limits their usefulness (13). (iii) Using WordPieces as tokens rather than words can improve the coverage of the input embeddings for the 1d-CNN classifier.

We further analyzed the errors made by our method. One kind of error is that the two entities in a relation are too far away to be identified by our method. For example, our method failed to extract the CID relation of ‘systemic sclerosis’ (MeSH: D012595) and ‘corticosteroid’ (MeSH: D000305) from the following sentence: ‘Scleroderma renal crisis (SRC) is a rare complication of **systemic sclerosis** (SSc) but can be severe enough to require temporary or permanent renal replacement therapy. Moderate to high dose **corticosteroid** use is recognized as a major risk factor for SRC*.’* (PMID: 22836123), the two entities (in bold) are separated in two sentences with 18 words between them. This may be because the distance between the entities is too far, and there is no trigger word between them.

Another kind of error is that the category of relations extracted by our method is not the category of relations annotated in the corpus. Take the BC5CDR corpus as an example, in ‘**Famotidine** is a histamine H2-receptor antagonist used in inpatient settings for prevention of stress **ulcers**...’ (PMID: 8701013, words in bold are disease/chemical entities given by the corpus), our method successfully extracted the relation of ‘Famotidine’ (MeSH: D015738) and ‘ulcers’ (MeSH: D014456) but cannot distinguish the difference between prevention relation and CID relation.

We trained our models on an NVIDIA GeForce GTX 1080Ti GPU with cuDNN library enabled. For the BC5CDR corpus, the average training speed is 0.126sec/batch, and the total time of model training is about 31 minutes. For the TCM corpus, take herb-disease relation as an example, the average training speed is 0.081sec/batch, and the training time for the model is about 13 minutes. For the i2b2 temporal corpus, the average training speed is 0.505sec/batch, and the total training time is about 50 minutes.

The results presented in the BioCreative V CDR task can improve the research process of chemical-disease interactions, which is critical in applications including clinical trial screening, clinical decision-making and drug discovery. The relations of herbs, formulae, syndromes and diseases found in TCM literature relation extraction task are useful for assisting clinical treatment, poly-pharmacology and drug-safety research. Understanding the clinical timeline is crucial in determining a patient’s diagnosis and treatment. The results presented in I2b2 2012 challenge clinical temporal relation extraction task can be used in disease progression monitoring, early prediction of chronic disease and adverse event detection.

## Conclusions

This paper has presented a novel approach to improve deep learning-based medical relation extraction via model pre-training on large-scale unstructured text and fine-tuning the pre-trained model. The approach employs BERT to construct a pre-trained model on large-scale unstructured text and uses 1d-CNN to fine-tune the pre-trained model for clinical relation extraction. We have conducted extensive experiments on three clinical relation extraction corpora in comparison with the best existing systems. Empirical results show that our approach achieves state-of-the-art performance on all three corpora. We have found that using the pre-trained model and fine-tuning technique boosts the performance of clinical relation extraction. This general approach can be applied to many disease- and drug-related systems and clinical applications.

## Notes

**Database URL**: http://120.78.238.14:8080/cdr
